# Optimized Fermentation Conditions of Pulses Increase Scavenging Capacity and Markers of Anti-Diabetic Properties

**DOI:** 10.3390/antiox14050523

**Published:** 2025-04-27

**Authors:** Andrea Jimena Valdés-Alvarado, Erick Damián Castañeda-Reyes, Elvira Gonzalez de Mejia

**Affiliations:** 228 ER Madigan Laboratory, Department of Food Science and Human Nutrition, University of Illinois, 2101 W. Gregory Dr, Champaign, IL 61801, USA

**Keywords:** antioxidant capacity, type 2 diabetes, fermentation, black eyed pea, green split pea, red lentil, *black bean*, pinto bean, pulses, *Lactiplantibacillus plantarum* 299v

## Abstract

Fermented pulses offer health benefits due to their antioxidant and antidiabetic properties. The objective was to optimize the fermentation conditions of black bean (BB), black eyed pea (BEP), green split pea (GSP), red lentil (RL), and pinto bean (PB), using *Lactiplantibacillus plantarum* 299v (Lp299v), based on the antioxidant-scavenging capacity and the ability to modulate type-2 diabetes markers. Pulses were grounded, dispersed in water, hydrolyzed with α-amylase, and pasteurized and inoculated with Lp299v. Optimization was performed by using the Box–Behnken response surface methodology, with the fermentation time, bacterial concentration, and flour concentration as variables. The values of antioxidant capacity measured as 2,2-diphenyl-1-picrylhydrazyl (DPPH)-radical scavenging of RL, BEP, PB, BB, and GSP were 57%, 68%, 71%, 72%, and 83%, respectively, under optimal conditions (8–9 h, 0.76–3.5 × 10^9^ a colony-forming unit (CFU)/mL, and 5.5–15 g flour/100 mL). These models demonstrated strong predictive power (*p* < 0.01) and a non-significant lack of fit (*p* ≥ 0.05). Additionally, fermentation increased the soluble protein content (3–10 mg/mL) and significantly inhibited dipeptidyl peptidase-IV and α-glucosidase activities by 40–70% and 30–60%, respectively. These results suggest that fermentation with Lp299v enhances the nutritional and functional quality of pulses, producing bioactive ingredients with antioxidant and antidiabetic potential. These functional ingredients may be used in the development of dietary interventions or as part of health-promoting foods, especially those targeted at the management of type-2 diabetes.

## 1. Introduction

Diabetes is recognized as a global epidemic, with approximately 589 million adults (20–79 years) affected and 3.4 million deaths in 2024, which is expected to rise to 853 million by 2050 [1]. This metabolic disease, characterized by chronic hyperglycemia and metabolic disruptions due to insulin inefficiency, manifests itself as type 1 diabetes, type-2 diabetes (T2D), and gestational diabetes. T2D comprises 90–95% of cases [2,3]. The pathophysiology of T2D involves progressive insulin resistance in peripheral tissues, accompanied by β-cell dysfunction and decreased insulin production, ultimately leading to glucose intolerance and metabolic complications [3]. While pharmaceuticals remain the most effective treatment for T2D, long-term medication use can cause various side effects [1,4]. However, recent research suggests that a diet rich in plant-based foods may help to prevent and manage T2D and other chronic diseases [5].

A plant-based diet with pulses increases fiber, protein, and mineral intake while reducing unhealthy fats, thereby enhancing the dietary quality through plant-based protein sources [6,7]. The American Dietary Guidelines recommend the consumption of 1 to 3 cups of beans, peas, and lentils as vegetable sources or 4 to 12 ounces of these pulses as protein sources per week [8]. Pulses are the dried edible seeds of legumes, including lentils, peas, and beans. In addition, protein derived from pulses (∼18–25% protein) provides high amounts of essential amino acids, such as lysine and leucine, crucial to preventing and treating T2D [4,9]. Proteins support cellular functions, including antioxidant activity, which reduces oxidative stress, maintains metabolism, and protects β-cells, which are vulnerable to ROS-induced insulin dysfunction due to low antioxidant levels [10,11,12,13]. Faba bean (FB) and lentil sprouts lower serum glucose through their flavonoid concentration, simultaneously improving liver enzyme levels and exhibiting hepatoprotective effects [14]. Pea proteins and their hydrolysis products possess biological activities and interact with the human gut microbiota, which influence antioxidant, antimicrobial, antihypertensive, and antidiabetic activities [15]. Therefore, a plant-based diet rich in pulses offers significant health benefits and disease prevention. Furthermore, processing methods such as soaking, germination, fermentation, and thermal applications enhance the nutritional quality of pulses by reducing antinutrients and boosting essential nutrients [16,17].

Among various methods, fermentation is an economical, energy-efficient technique that increases nutrients, produces bioactive compounds, and reduces antinutritional factors [18]. Fermentation improves the solubility of proteins in water and increases digestibility by breaking down non-nutritional factors and enabling enzymatic crosslinking. Fermentation also generates bioactive peptides that regulate glucose metabolism and remain stable during digestion, making fermented products valuable for health improvement [19,20]. The outcome of fermentation can vary depending on the type of pulse and microorganism used. Fermented black bean (BB) modulates markers related to T2D and obesity, such as α-amylase and α-glucosidase (AG) inhibition, possesses antioxidant capacity, and reduces lipid accumulation due to its bioactive components, including peptides [21]. FB fermented with *Lactiplantibacillus plantarum* (*L. plantarum*) VTT E-133328 improved in vitro protein digestibility [22]. As noted by Nordström et al. [23] and Kaźmierczak-Siedlecka et al. [24], *L. plantarum* 299v (Lp299v) survives the passage through the gastrointestinal tract despite gastric acidity, supports immunity, reduces inflammation, enhances iron absorption, and inhibits pathogens. Additionally, fermenting pulse-based beverages, such as those made from lupin, chickpea, red lentil, and green lentil, with Lp299v has been shown to enhance antioxidant properties, significantly increasing total phenolic and flavonoid concentrations [25,26]. Peptides from fermented pulses may help to manage T2D by enhancing insulin secretion, increasing insulin sensitivity, and inhibiting glucose-metabolism enzymes, such as dipeptidyl peptidase IV (DPP-IV), α-amylase, and AG, which highlights their anti-diabetic and anti-inflammatory effects [27].

In this study, we hypothesized that optimizing the fermentation conditions of pulses increases their bioactive potential depending on their original chemical composition. Therefore, the aim was to enhance antioxidant and anti-diabetic potential by optimizing the fermentation process of red lentil (RL), black-eyed pea (BEP), green split pea (GSP), black bean (BB), and pinto bean (PB) by using Lp299v as the fermenting microorganism. We also focused on analyzing the protein profiles, antioxidant capacity based on 2,2-diphenyl-1-picrylhydrazyl (DPPH) and nitric oxide (NO) scavenging capacity, and the biochemical inhibition of enzymes, such as DPP-IV, and AG, which are relevant to T2D management.

## 2. Materials and Methods

### 2.1. Materials

Five types of pulses, RL (*Lens culinaris)*, BEP (*Vigna unguiculata*), GSP (*Pisum sativum*), BB (*Phaseolus vulgaris*), and PB (*Phaseolus vulgaris*), were provided by Dr. Dave Luthria from the US Department of Agriculture, purchased from local markets to ensure that they were products available to consumers. The raw pulses were ground on a commercial grinder (7 L, Robot Coupe, Blixer 6VV, Ridgeland, MS, USA) and stored in zip lock bags at 4 °C until use. *L. plantarum* 299v (Lp299v) was purchased from GoodBelly Probiotics (Boulder, CO, USA). The enzymes used α-amylase type VI-B was from the porcine pancreas (EC 3.2.1.1, 10.3 U/mg solid) and AG was from *Saccharomyces cerevisiae* (EC 3.2.1.20), and DPPH was purchased from Sigma–Aldrich (St. Louis, MO, USA). High-purity α-amylase from porcine pancreas (E-PANAA; 75,000 U/g) was purchased from NEOGEN (Lansing, MI, USA). Human dipeptidyl peptidase IV (228-10340-2) was obtained from RayBiotech (Peachtree Corners, GA, USA). An RCDC protein assay kit, a modified form of the Lowry assay that is both reducing-agent-compatible (RC) and detergent-compatible (DC), gel electrophoresis gels, and standard MW dual color were purchased from Bio-Rad (Hercules, CA, USA). Gly-Pro-p-nitroanilide (21244) was purchased from Cayman Chemicals (Ann Arbor, MI, USA). The antibody GAPDH (human sequence) was obtained from Santa Cruz Biotechnology (Dallas, TX, USA). The antibodies PLCβ2 and SGLT1, as well as 2-(*N*-(7-nitrobenz-2-oxa-1,3-diazol-4-il)amino)-2-deoxyglucose (2-NBDG), were purchased from Invitrogen (Carlsbad, CA, USA). The antibody Glut2 was obtained from Sigma Millipore (St. Louis, MO, USA). The secondary Amersham ECL-HRP-conjugated antibody, and Amersham Hybond P 0.45 PVDF blotting membrane were obtained from Cytiva (Marlborough, MA, USA). Clarity Western ECL substrate was purchased from Bio-Rad (Hercules, CA, USA). All other reagents were obtained from Sigma–Aldrich (St. Louis, MO, USA).

### 2.2. Fermentation of Pulses with Lp299v

**Figure 1:**
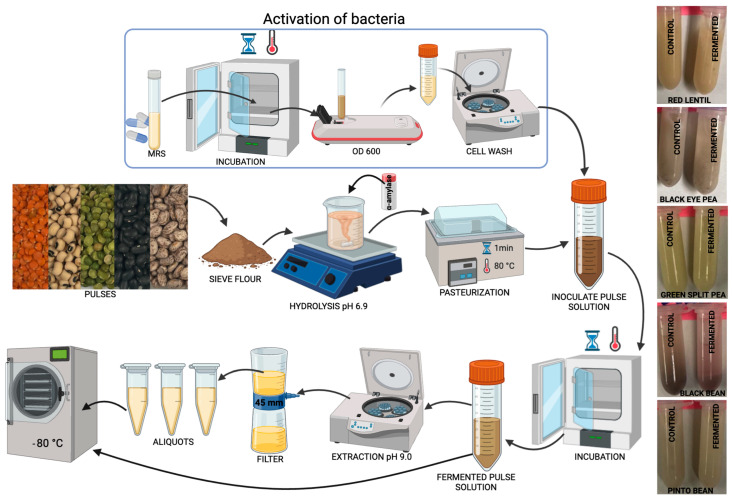
Diagram of pulse fermentation.

In the first step, 15% pulse flour was added to distilled water (*w*/*v*), and the pH was adjusted to 6.9 by using 6 M sodium hydroxide (NaOH). While the mixture was stirred, commercial α-amylase was incorporated at one unit per mg of starch, according to the manufacturer’s instructions, and stirred for 2 h at 20 °C. α-Amylase was used to reduce the viscosity by hydrolyzing starch, thereby improving the texture and facilitating processing and fermentation. The pulse homogenate was pasteurized in a water bath at 80 °C for 1 min to reduce native microorganisms. Then, the initial 15% pulse homogenate was diluted in water to obtain 3% and 9% concentrations. Once cooled to 37 °C, to prevent thermal stress, which could have compromised bacterial viability, the homogenate was inoculated with viable Lp299v cell counts of approximately 5 × 10^8^, 2 × 10^9^, and 3.5 × 10^9^ colony-forming units (CFU) per mL and mixed at low shear. The viable bacteria were counted by using the plate-counting method on De Man–Rogosa–Sharpe (MRS) agar, involving serial dilutions of the suspension and subsequent incubation at 37 °C for 24 h to allow for colony formation. The controls were prepared in the same way but without the addition of Lp299v, referred to as the control (no Lp299v). The pulse homogenate was incubated at 37 °C in closed containers under constant shaking (300× *g*) until the specified time was reached (8 h, 16 h, or 24 h). After fermentation, the pH was recorded to assess the efficiency of the process. For soluble protein extraction from the fermented and control (no Lp299v) samples, the pH was adjusted to 9.0, stirred for 1 h, centrifuged (3214× *g* for 40 min), filtered (0.45 µm, low protein binding), and stored at −20 °C for further analysis (Figure 1).

The response surface methodology (RSM) was employed to determine the conditions under which the fermented sample exhibited the highest antioxidant-scavenging capacity and thus optimize the fermentation of pulses. The independent input variables, included the fermentation time (X_1_), the CFU of bacteria (X_2_), and flour concentration (X_3_). The values used for optimization were coded levels of −1, 0, and +1; X_1_ represented 8, 16, or 24 h; X_2_ represented 5 × 10^8^, 2 × 10^9^, or 3.5 × 10^9^ CFU/mL; and *X*_3_ represented 3, 9, or 15% pulse flour-water (*w*/*v*). The output variable of the optimization for each pulse was the antioxidant activity based on the DPPH method. A second-order polynomial model was applied to predict the optimal conditions. The regression equation was as follows:

(1) $$Y=\beta_{0}+\beta_{1}X_{1}+\beta_{2}X_{2}+\beta_{3}X_{3}+\beta_{12}X_{1}X_{2}+\beta_{13}X_{1}X_{3}+\beta_{23}X_{2}X_{3}+\beta_{11}X_{1}^{2}+\beta_{22}X_{2}^{2}+\beta_{23}X_{3}^{2}$$

In the proposed model, Y represents the predicted outcome and percentage of scavenging capacity based on DPPH. The model’s constant is denoted as β_0_, and X_1_, X_2_, and X_3_ represent the independent predictors: fermentation time, CFU of bacteria, and flour concentration, respectively. The coefficients β_1_, β_2_, and β_3_ correspond to the squared terms, highlighting the model’s quadratic nature. The coefficients β_12_, β_13_, and β_23_ indicate the interactions between the predictors. Furthermore, β_11_, β_22_, and β_33_ are the coefficients for the individual squared predictors.

### 2.3. Antioxidant Assays

#### 2.3.1. Measurement of 2,2-diphenyl-1-picrylhydrazyl (DPPH)-Radical-Scavenging Capacity

The DPPH capacity of RL, BEP, and GSP was analyzed according to Fonseca-Hernández et al. [28] and Huang et al. [29], with some modifications. Under light-protected conditions, in a 96-well plate, 25 μL of the filtered sample was mixed with 175 μL of a 0.1 mM DPPH solution prepared in 70% methanol. After shaking, the mixture was incubated for 2 h, and absorbance was measured at 517 nm. A 200 μM Trolox solution was used as a scavenging-capacity control to assess the assay efficiency. The DPPH-radical-scavenging capacity was quantified and reported as a measure of the antioxidant capacity.

The DPPH activity of BB and PB was analyzed following the method described by Grancieri et al. [30] and Huang et al. [29], which allows for better pigment separation. Briefly, 100 μL of the filtered sample and 1.5 mL of methanolic DPPH solution were added to a 1.7 mL tube. The mixture was then vortexed and incubated in the dark for 25 min. The tubes were centrifuged for 5 min at 20,000 × *g*. Then, 200 μL of supernatant was added to a 96-well plate, and the absorbance was measured at 517 nm. A 200 μM Trolox solution served as the reference control to evaluate the efficiency of the assay’s scavenging capacity.

#### 2.3.2. Measurement of Nitric Oxide (NO)-Scavenging Capacity

Nitric oxide production was adapted from Grancieri et al. [30]. This method measures the accumulation of nitrite (NO_2_), a stable byproduct formed when NO reacts with oxygen in an aqueous solution. A 300 µL aliquot of 50 mM sodium nitroprusside was combined with 300 µL of the sample or PBS (0.1 M, at pH 7.4, as the blank) for 90 min at room temperature. After incubation, 300 µL of Griess reagent was added to each well and incubated for an additional 10 min. The tubes were then centrifuged for 5 min at 20,000 × *g*. Finally, 200 μL of supernatant was added to a 96-well plate, and the absorbance was measured at 550 nm. L-Ascorbic acid (0.1 M) was used as the control. The concentration of the NO-radical-scavenging capacity was calculated.

### 2.4. Protein and Peptide Profile

#### 2.4.1. Protein Quantification

The determination of soluble protein in the filtered pulses with a pH of 9.0 was conducted in triplicate by using the protein RCDC assay, compatible with reductants and detergents, by Bio-Rad, following the manufacturer’s instructions. Briefly, 25 μL of the fermented sample or control (no Lp299v), previously diluted (1:10, *v*/*v*), and 125 μL of RC reagent I (reducing agent) were placed in a microcentrifuge tube and vortexed. Then, 125 μL of RC reagent II (reducing agent) was added and centrifuged. After discarding the supernatant, 127 μL of reagent A’ (alkaline copper tartrate solution mixed with surfactant solution) was added and vortexed. After 5 min of incubation, 1 mL of reagent B (Folin reagent) was added and incubated for 15 min. Finally, 200 μL was added to each well, and the absorbance was read at 700 nm. The concentration was calculated by using a bovine serum albumin (BSA) standard curve (y = 0.0002x − 0.0445, R^2^ = 0.99).

#### 2.4.2. Protein Profile Based on Gel Electrophoresis Analysis (SDS-PAGE)

Gel electrophoresis was conducted to visualize the protein profile according to the manufacturer’s guidelines. The fermented pulse or control (no Lp299v) homogenate (40 μg of protein in 15 μL) was 1:1 (*v/v*) diluted in Laemmli buffer containing 5% β-mercaptoethanol and boiled for 5 min. Then, 15 μL was loaded onto sodium dodecyl sulfate-polyacrylamide gel for electrophoresis (SDS-PAGE, 4–20%). A standard MW dual color marker (10–250 kDa) was used to estimate the molecular mass of the separated proteins. The gels were run at 170 V for 35 min, washed with distilled water for 10 min, and left to shake with Simply Blue Safe Stain (Invitrogen, Carlsbad, CA, USA) for 1h. The gels were washed with distilled water overnight. Images were acquired by using an Amersham ImageQuant 800 Fluor (Cytiva, Marlborough, MA, USA).

#### 2.4.3. Proteolytic Activity Determination of Commercial and High-Purity α-Amylase

The proteolytic activity of commercial and high-purity α-amylase was determined following the method by Merheb-Dini et al. [31]. This assay was performed because gel electrophoresis showed changes in the protein profile after “commercial” α-amylase hydrolysis (10.3 U/mg) in comparison with higher-purity α-amylase (75 U/mg), since α-amylase may have contained protease contaminants. Briefly, 50% (*w*/*v*) casein in 0.5 M NaOH was further diluted in 0.2 M acetate buffer (pH 5.5) to obtain a 0.5% solution. Then, 0.4 mL of the 0.5% solution was mixed with 0.4 mL of 0.2 M acetate buffer in microcentrifuge tubes. Subsequently, 0.2 mL of either 1% (*w*/*v*) commercial α-amylase or 1% (*v*/*v*) high-purity α-amylase, both prepared in 0.2 M acetate buffer, was added to the mixture. The reaction was incubated at 35 °C for 30 min and then terminated by adding 1 mL of 10% trichloroacetic acid (TCA). After centrifugation at 2300× *g* for 5 min, the absorbance of the resulting supernatant was measured at 280 nm. A control sample was prepared by adding TCA before introducing the enzyme solution. Proteolytic activity was calculated as described by Merheb-Dini et al. [31].

#### 2.4.4. LC-MSMS Peptide Profile

The peptides in soluble protein were analyzed with LC-ESI-MSMS following the methods of Acevedo-Martínez and de Mejia [32] and Kusumah et al. [33], with some modifications. Briefly, dilutions in water were made to obtain a 2 mg/mL protein concentration and the solutions were analyzed with a Q-ToF Ultima mass spectrometer (Waters, Milford, MA, USA), equipped with an Alliance 2795 HPLC system. The separation of the components was performed by using gradient mobile phase A (95% water, 5% acetonitrile, and 0.01% formic acid) and gradient mobile phase B (95% acetonitrile, 5% water, and 0.1% formic acid) at a flow rate of 400 μL/min for 15 min in total, and a PDA detector wavelength of 280 nm. MassLynx 4.1 V software (Waters, Milford, MA, USA) was used for instrument control and to obtain the chromatograph for the further comparison of the peptide profiles.

### 2.5. T2D Markers

#### 2.5.1. DPP-IV Inhibition

The methodology described by Di Stefano et al. [34] and Lacroix and Li-Chan [35] was employed with slight modifications for the DPP-IV-inhibition analysis. Initially, a 96-well microplate assay was established where 25 µL of the filtered fermented sample and 25 µL of the substrate Gly-Pro-p-nitroanilide (2 mM) were preincubated at 37 °C for 10 min. Subsequently, 50 µL of the DPP-IV enzyme (0.02 unit/mL) was added, and the mixture was incubated at 37 °C for 30 min. The absorbance of the released p-nitroanilide was measured at 405 nm. The enzyme activity without inhibitor and using Tris-HCl buffer (100 mM, at pH 8) served as a positive control. The negative control was prepared with Tris-HCl buffer instead of the sample and enzyme. Sample blanks were created with the sample, substrate, and Tris-HCl buffer. Sitagliptin was also utilized as a reference inhibitor.

#### 2.5.2. α-Glucosidase Inhibition

AG was assayed as described by Chandrasekaran and de Mejia [36]. Briefly, 50 μL of the filtered fermented sample or positive control (1 mM acarbose) was added to 100 μL of AG (0.5 U/mL) in a 96-well plate. After incubation for 10 min at 25 °C, 50 μL of p-nitrophenyl-α-D-glucopyranoside (5 mM) was added. The reactions were incubated for 5 min at 25 °C. The absorbance was measured at 405 nm by using a microplate reader and compared with a control that contained 0.1 M sodium phosphate buffer at pH 6.9 instead of the sample.

### 2.6. In Vitro Assays

The freeze-dried fermented pulses were resuspended in phenol-red-free, glucose-free medium at a concentration of 300 mg fermented pulse/mL, sonicated for 60 min in a water bath sonicator, and centrifuged for 30 min at 4000× *g*; then, the supernatant was filtered through 45 μL syringe filters, and the filtrate was used for the treatments.

#### 2.6.1. Differentiated Caco2 Cells

Caco-2 cells were seeded at 5 × 10^4^ cells/well in 96-well plates and at 1 × 10^6^ cells/well in 6-well plates. The cells were cultured with Dulbecco’s Modified Eagle Medium (DMEM) supplemented with 10% fetal bovine serum (FBS) and 1% antibiotics, and the monolayers were allowed to differentiate for 21 days and were maintained at 37 °C with 5% CO_2_. The medium was changed every other day during the differentiation period [37].

#### 2.6.2. Glucose Uptake, Expression of Glucose Absorption-Related Markers Based with Western Blot, and DPP-IV Inhibition

Glucose uptake was determined with the 2-NGBD assay [38]. Caco-2 cells were allowed to differentiate as previously described and treated with 100 μM phloretin and the fermented pulse at different concentrations (1, 3, and 30 mg RLF/mL) resuspended in glucose-free medium. After 24 h (22 days since the beginning of the differentiation process), the cells were treated with 100 μM 2-NGBD in a glucose-free medium for 1 h, followed by three washes with phosphate-buffered saline (PBS) to stop the glucose uptake process and read in a Synergy2 multi-well plate reader (Biotek, Winooski, VT, USA), and their absorbance was read at λ_ex_ 485 nm and λ_em_ 535 nm.

The differentiated Caco-2 cells seeded in 6-well plates were used for producing the lysates. For gel electrophoresis, the protein contents in the lysates were measured by using the DC protein assay following the manufacturer’s protocol and adjusted to 20 μg of protein/well. For the Western blots, the protein was transferred to PVDF membranes using a Bio-Rad mini Trans-Blot^®^ cell system at 100 mV for 1 h in a cold room at 4 °C. Once transferred, the membrane was blocked using 5% non-fat dry milk, followed by five washes of 5 min each with Tris Buffered Saline with Tween 20 (TBST). The primary antibodies SGLT1 (1:1000), GAPDH (1:1000), Glut2 (1:1000), and PLCβ2 (1:1000) were applied overnight and followed by five washes with TBST, and incubation for 1 h at room temperature with the secondary antibody linked to HRP. Clarity Western ECL substrate was used to reveal the protein bands with an ImageQuant 800 System (GE Healthcare, Buckinghamshire, UK) [39].

Differentiated Caco-2 cells were treated with 100 μM phloretin (Glut-2 inhibitor) and phlorizin (SGLT1 inhibitor) and the fermented pulse at different concentrations (0, 1, 3, and 30 mg/mL) to determine DPP-IV inhibition. After a 24 h treatment, the medium was removed, and the substrate namely Gly-Pro-p-nitroalinide (100 μM) was added. The absorbance was read at 405 nm after a 60 min of incubation [39].

### 2.7. Statistical Analysis

Design Expert 12 software (Stat-Ease Inc., Minneapolis, MN, USA) was used to design, screen, and optimize the conditions of the fermented samples to achieve the highest antioxidant properties, as well as creating the 3D response surface plots and performing the regression analysis. The experiments, after optimization, were conducted in quadruplicate. Data are expressed as the mean ± standard deviation or standard error for RSM. The statistical analyses were performed by using ANOVA followed by a Tukey’s test and paired t-test for the comparison of experimental (fermented with Lp299v) and control (no Lp299v) values and the correlations between assays for each experimental group and the control in the program GraphPad Prism 10.3.1. In the in vitro studies, ANOVA followed by Tukey’s multiple comparison test was used. The results are reported as the mean ± SD of independent repetitions; differences were considered significant at *p* < 0.05.

## 3. Results and Discussion

This research demonstrated the optimized antioxidant properties of pulse substrates (RL, BEP, GSP, BB, and PB) fermented with Lp299v. As reported by various authors, the bioactive compounds generated during fermentation depend on both the type and concentration of the substrate and the bacterial strain used.

### 3.1. Fermentation Kinetics Based on pH Changes

The fermentation kinetics of the different pulses and conditions were evaluated according to the pH (Table 1). The final pH in all the fermented pulses decreased significantly (*p* ≤ 0.05) from the initial pH of 6.9. Although there were slight variations in the pH across the samples, there was no significant (*p* ≥ 0.05) change in the final pH when grouped by flour concentration, the CFU of Lp299v, or time. The final pH values of the fermented pulse homogenates were approximately 3.8, 3.8, 3.7, 3.9, and 3.8 for RL, BEP, GSP, BB, and PB, respectively. This indicates that Lp299v can metabolize readily fermentable compounds and acidify pulses in 8 h of fermentation, which may be considered a suitable substrate for the growth of this bacteria. Previous studies support these findings. In Hurtado-Murillo et al. [40], fermented milk-like beverages of chickpea and quinoa reached a pH of 4.3 after 8–10 h of lactic acid fermentation. Skrzypczak et al. [26] documented that the pH of RL, green lentils, and chickpeas fermented with Lp299v for 48 and 72 h had a decrease in pH from 7.6 to 5.3, from 7.0 to 5.5, and from 7.5 to 4.8, respectively. They also suggested that, beyond the acidification caused by LAB, the bacterial proteolytic system influenced the pH by releasing alkaline by-products from protein breakdown, which could modulate the extent of acidification. Huang et al. [41] reported that LAB produce proteases that allow for protein utilization, enhancing fermentation abilities due to the growth of LAB, organic acid production, and a reduction in the pH. Additionally, enzymatic activity, such as the hydrolysis of starch by α-amylase, may accelerate acid production by generating simple sugars as fermentation substrates [42]. Although LAB are equipped with defense and adaptation mechanisms, extremely low pH can disrupt the LAB physiological process, leading to an obstruction of growth and promoting cell mortality because the bacteria struggle to maintain a stable intracellular pH [43].

### 3.2. Optimized Fermentation Conditions

Table 1 presents the DPPH-scavenging capacity as the output variable to indicate the antioxidant capacity based on the conditions used in the RSM for pulse fermentation. The results of the *p*-value ANOVA are summarized in Table 2. The RSM evaluates the effect of the time, and bacteria, and pulse flour concentrations, as well as their interactions and the quadratic terms, on the response variables. The models were statistically significant (*p* ≤ 0.05) for all the pulses and therefore adequate for prediction within the range of variables employed.

The factor “X_1_-Time” suggested a strong linear influence on the response variable, as the quadratic parameters were not statistically significant (*p* ≥ 0.05). The factor “X_2_-Bacteria” had a *p*-value higher than 0.05, meaning that the CFU of bacteria did not have a significant effect on the fermentation of each pulse. The factor “X_3_-Flour concentration” showed a significant impact on RL, BEP, GSP, and PB but not on BB.

The interaction and quadratic effects were not significant (*p* ≥ 0.05) for any of the pulses. However, the quadratic effect of “X_3_-Flour concentration” was significant for RL, BEP, BB, and PB, suggesting a nonlinear influence of this factor on the DPPH-scavenging activity of the fermented pulses. GSP did not show a quadratic effect since it did not show sufficient nonlinearity, meaning that it did not significantly improve the model. Therefore, a two-factor interaction model was used.

**Figure 2:**
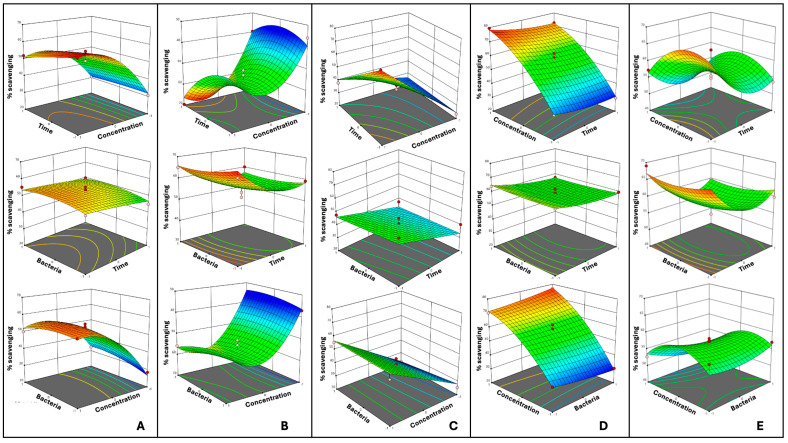
Response surface plots for the optimization of fermentation of pulses affected by the fermentation time, CFU of Lp299v, and flour concentration (flour in water, *w*/*v*) in terms of the response variable scavenging capacity (%) based on DPPH. (**A**) Red lentil, (**B**) black eyed pea, (**C**) green split pea, (**D**) black bean, and (**E**) pinto bean. Red dots indicate design points above predicted values, and yellow dots indicate design points below predicted values.

The lack of fit was not significant (*p* ≥ 0.05) for any of the pulses, indicating that the models adequately fit the data. Figure 2A–E show each pulse’s three-dimensional response surface plots. As presented, the nonlinearity of the interactions can be seen as U-shaped or inverted U-shaped curves. However, GSP (Figure 2C) did not show a quadratic effect, meaning that its response surface did not display enough curvature to justify quadratic terms in the model. The predicted and observed DPPH scavenging capacity under the suggested and tested optimized values are presented in Table 3. After the evaluation of the 16 different conditions for each pulse, a multiple regression analysis was applied to find the relationship between the independent variables (X_1_, X_2_, and X_3_) and the dependent variables (the percentage of scavenging capacity based on the DPPH assay) with a second-degree polynomial equation with the following findings:

Equation for RL

(2) $$Y=3.2879+0.2021X_{1}+11.9928X_{2}+7.8817X_{3}+0.0721X_{1}X_{2}-0.04135X_{1}X_{3}-0.5467X_{2}X_{3}-0.0067X_{1}^{2}\;-3.6089X_{2}^{2}-0.2602X_{3}^{2}$$

Equation for BEP

(3) $$Y=30.8896-2.6079X_{1}-3.2185X_{2}+10.2895X_{3}+0.0987X_{1}X_{2}-0.0961X_{1}X_{3}-0.5384X_{2}X_{3}\;+0.086937X_{1}^{2}+2.4886X_{2}^{2}-0.3739X_{3}^{2}$$

Equation for GSP

(4) $$Y=14.4339+0.4939X_{1}-6.8792X_{2}+5.0736X_{3}+0.1461X_{1}X_{2}-0.1755X_{1}X_{3}+0.3364X_{2}X_{3}$$

Equation for BB

(5) $$Y=85.3722-3.6644X_{1}-2.74833X_{2}+1.7244X_{3}-0.03542X_{1}X_{2}+0.0462X_{1}X_{3}-0.3317X_{2}X_{3}+0.0901X_{1}^{2}\;+2.5933X_{2}^{2}{-0.1382X}_{3}^{2}$$

Equation for PB

(6) $$Y=33.0135-1.1024X_{1}-7.0817X_{2}+5.8201X_{3}-0.1096X_{1}X_{2}-0.0007X_{1}X_{3}+0.6844X_{2}X_{3}+0.0263X_{1}^{2}\;+1.2000X_{2}^{2}-0.1698X_{3}^{2}$$

where the linear terms X_1_ represent the fermentation time (hours), X_2_ represents the bacteria log CFU/mL of Lp299v, and X_3_ represents the flour concentration level (% flour in water, *w/v*).

The interaction terms account for the combined effect of two variables (e.g., X_1_X_2_ represents the interaction between time and bacteria).

The quadratic term (i.e., X_1_^2^) allows the model to detect the non-linear effect of a variable.

**Table 3:** Optimization and verification of the model of pulse fermentation conditions for DPPH activity.

	Factor			DPPH (%)		Final pH
	X_1_: Time (h)	X_2_: Bacteria (CFU/mL)	X_3_: Flour Concentration (g/100 mL)	Predicted	Observed Verification	Observed Condition
**RL**	9	1.34 × 10^9^	13.6	60.44 ± 2. 43	56.65 ± 2.84	4.07 ± 0.00
**BEP**	8	1.70 × 10^9^	11.0	69.74 ± 4.01	67.77 ± 1.47	3.88 ± 0.02
**GSP**	8	0.76 × 10^9^	15.0	73.12 ± 4.45	82.67 ± 6.03	4.02 ± 0.02
**BB**	8	3.50 × 10^9^	5.5	68.61 ± 3.01	72.35 ± 5.48	3.95 ± 0.02
**PB**	8	2.58 × 10^9^	14.9	79.61 ± 1.90	70.87 ± 3.68	3.90 ± 0.00

Note: means ± standard deviations from three replicates. RL = red lentil; BEP = black eyed pea; GSP = green split pea; BB = black bean; PB = pinto bean. The final pH of the controls (no Lp299v added) was RL = 5.68 ± 0.05, BEP 6.34 ± 0.06, GSP = 5.63 ± 0.00, BB = 5.76 ± 0.10, and PB = 5.62 ± 0.05.

### 3.3. DPPH-Scavenging Capacity

**Figure 3:**
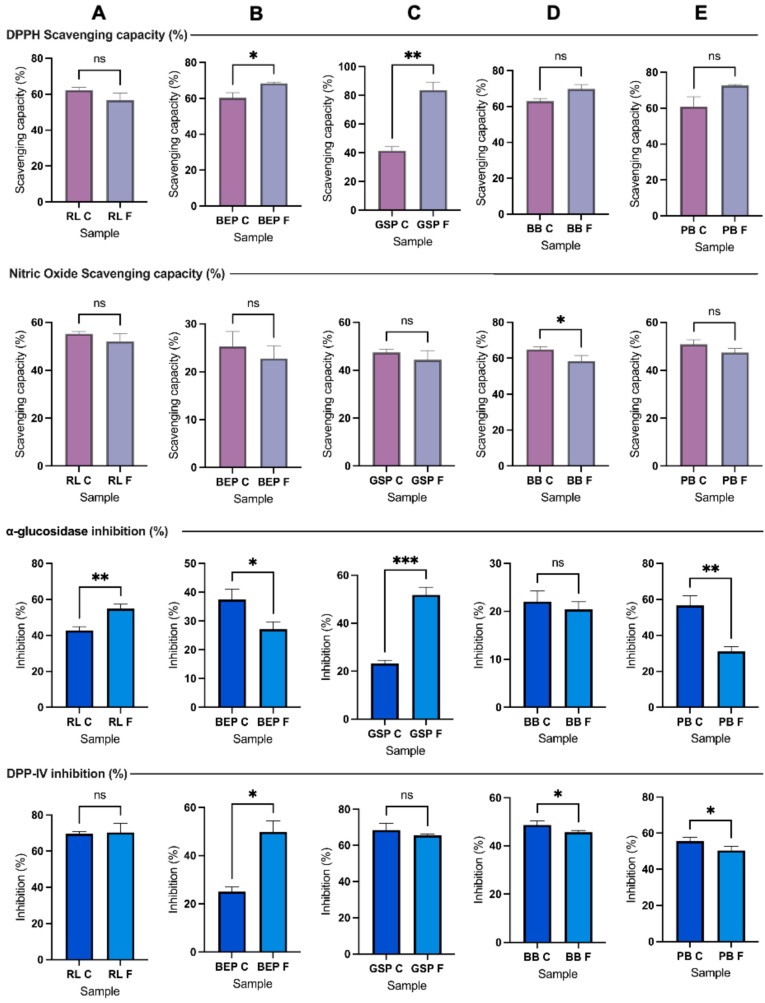
Scavenging antioxidant capacity and markers of anti-diabetic activity. Columns: (**A**) RL = red lentil, (**B**) BEP = black eyed pea, (**C**) GSP = green split pea, (**D**) BB = black bean, and (**E**) PB = pinto bean. RL C, BEP C, GSP C, BB C, PB C = controls, RL F, BEP F, GSP F, BB F, PB F = fermented; ns = not statistically different (*p* ≥ 0.05), * *p* ≤ 0.05, ** *p* ≤ 0.01, *** *p* ≤ 0.001.

Compared with the controls (no Lp299v fermentation), the scavenging capacity of the fermented BEP, GSP, BB, and PB was significantly higher (*p* ≤ 0.05) (Figure 3). Transformations during fermentation, such an increase in the phenolic concentration, bioactive peptides, organic acids, and vitamins, is responsible for the variations in the antioxidant activity [44]. Likewise, the findings are comparable to those reported by Liang et al. [45] who observed that the antioxidant capacity of lactic acid fermented mung bean increased over time, which might be attributed to the degradation of proteins to small peptides, isoflavones, and the production of lactic acid. However, the fermented RL revealed the lowest scavenging capacity compared with the other pulses and the control (no Lp299v). Mousavi et al. [46] reported that raw buckwheat and lentil exhibited inherent antioxidant activity with scavenging capacities of 19.8% and 25.2%, respectively, and after fermentation, the optimized beverage resulted in a 41.0% scavenging capacity. Similarly, Skrzypczak et al. [26] also found an increase of 26% in the radical-scavenging capacity after 72 h of fermentation. Moreover, Ali et al. [47] stated that the antioxidant power could be affected depending on the biological process and strain used to ferment the beans.

### 3.4. Nitric Oxide (NO)-Scavenging Capacity

As illustrated in Figure 3, the pulses RL, BEP, and GSP did not show a significant difference in NO (*p* ≥ 0.05) compared to their control (no Lp299v). However, the fermented BB and PB exhibited a significantly lower NO difference (*p* ≤ 0.05) than the control. Similarly, Zhao et al. [48] demonstrated that different compounds tested from barley extracts fermented with LBPs, such as saccharides, amino acids, nucleosides, and some organic acids, significantly decreased over time, specifically at 8 h. For instance, phenolic compounds, such as malic acid, vanillin, and gallic acid, were consumed due to physiological and metabolic processes [49,50].

Additionally, the fungal fermentation time of lentils affected their phenolic composition [50]. With the study of fermented lentils, we also found that the antioxidant activity during fermentation varied (increasing, decreasing, or remaining stable) depending on the assay used (DPPH, hydroxyl free radical scavenging activity, reducing power activity, and total antioxidant capacity) and the duration of fermentation. Therefore, further studies are needed to evaluate the effects of fermentation on the compounds consumed or produced and the appropriate methods to evaluate the biological activities under liquid-state fermentation. Specifically, research should focus on how these changes influence NO and DPPH assays. This would provide insights into the potential in vitro and further in vivo applications and the implications for oxidative stress and antioxidant activity.

NO is a molecule involved in various physiological processes, including vascular homeostasis, neurotransmission, platelet activation, intracellular and extracellular signaling, the prevention of oxidative stress and cytokine activation, and the acceleration of wound healing [50,51,52]. However, high concentrations of NO can also lead to tissue damage and cellular death. Consequently, antioxidants are utilized by the metabolism to balance excessive reactive nitrogen species, such as NO, and counteract oxidative stress [52,53].

### 3.5. Protein Profiles and Quantification

**Figure 4:**
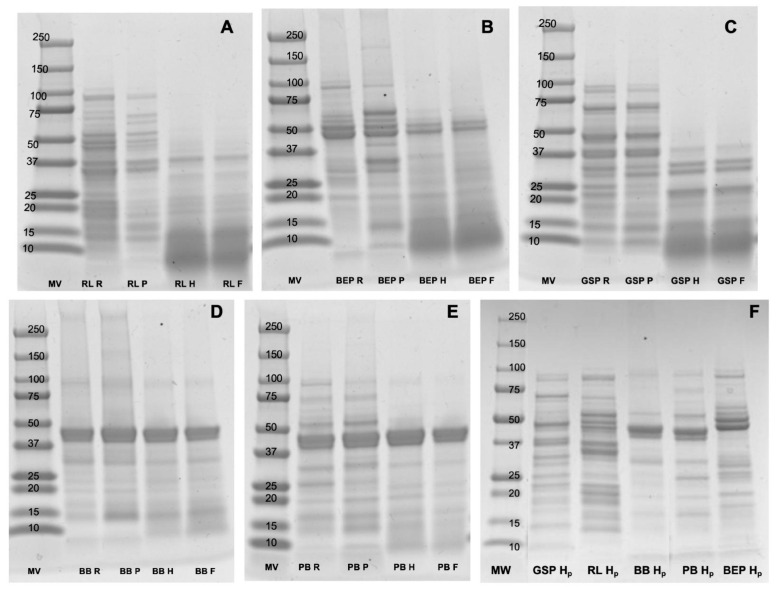
Comparison of protein profiles of pulses obtained with SDS-PAGE. (**A**) RL = red lentil, (**B**) BEP = black eyed pea, (**C**) GSP = green split pea, (**D**) BB = black bean, (**E**) PB = pinto bean, (**F**) All pulses were hydrolyzed with high-purity α-amylase. MV = ladder, R = raw, P = pasteurized, H = hydrolyzed with commercial α-amylase, F = fermented, and H_P_ = hydrolyzed with pure α-amylase.

Figure 4 shows a comparison of the protein profiles for the different pulses. The main subunits of the storage proteins from RL (70 kDa and 50 kDa subunits), BB, and PB (40 kDa and 20 kDa subunit and 47 kDa subunit), GSP (40 kDa and 20 kDa, and ∼47 kDa, ∼50 kDa, ∼34 kDa, and ∼30 kDa subunits), and BEP (strong bands at 50 kDa and 47 kDa) were observed. A clear distinction was seen between the pulses that were not hydrolyzed (RL R, BEP R, GSP R, BB R, PB R; RL P, BEP P, GSP P, BB P, and PB P) and those that were hydrolyzed with commercial α-amylase (RL H, BEP H, GSP H, BB H, and PB H) (Figure 4A–E). In contrast, no significant differences in protein profiles were evident between the control (no Lp299v) and fermented pulses (RL F, BEP F, GSP F, BB F, and PB F; Figure 4F), though a few bands in the fermented pulse profile appear to be either lost or faint, suggesting the presence of smaller polypeptides, possibly below 10 kDa. Similar results were obtained by Emkani et al. [54], who reported that the proteolytic activity and-acid-production capacity of bacteria depend on the strain; for instance, lactic acid bacteria metabolize proteins from pea, leading to the formation of smaller peptide chains and free amino acids.

**Figure 5:**
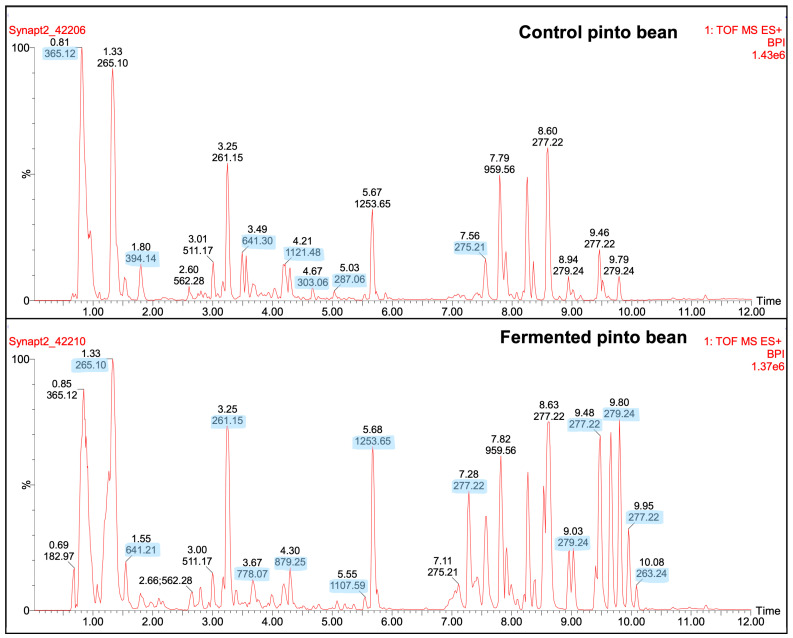
Representative results of LC-ESI-MSMS showing peaks of fermented pinto bean in comparison with the control (no Lp299v). Distinct peaks correspond to various peptides detected in the pulses. Blue highlights an increase in the intensity at specific *m*/*z* values.

A further qualitative analyses of peptide peaks from the control (no Lp299) and fermented samples were conducted by using LC-ESI-MSMS and the number of peaks, their presence or absence after fermentation, and their general profiles were determined. Some of the peaks in the fermented BEP, GSP, PB, and BB exhibited increased intensities compared with the control; however, the fermented RL did not show higher intensities than the control (Figure 5, Supplementary Figure S1). As shown in Figure 5 and Supplementary Figure S1, around 7 to 14 peaks increased in intensity in the fermented samples. Future studies should focus on sequencing and identifying these peptides to elucidate their composition and potential bioactive properties.

Proteolytic activity determination for α-amylase was conducted by using the methodology described by Merheb-Dini et al. [31]. The commercial α-amylase, as received from the provider, showed a proteolytic activity of 0.12 U/mL or 0.58 U/mg solid. In contrast, the highly pure α-amylase did not show proteolytic activity. Figure 4F illustrates the protein profile of the different pulses after hydrolysis using high-purity α-amylase. No obvious changes were detected in the protein profiles obtained with SDS-PAGE among the conditions raw, pasteurized, and hydrolyzed with the high-purity α-amylase. However, noticeable differences were observed when comparing these samples with those hydrolyzed with the commercial α-amylase and the fermented product. The commercial α-amylase was chosen due to its accessibility, cost, and wide use in the food industry. In addition, the hydrolyzed protein remained in the extract to be fermented.

**Figure 6:**
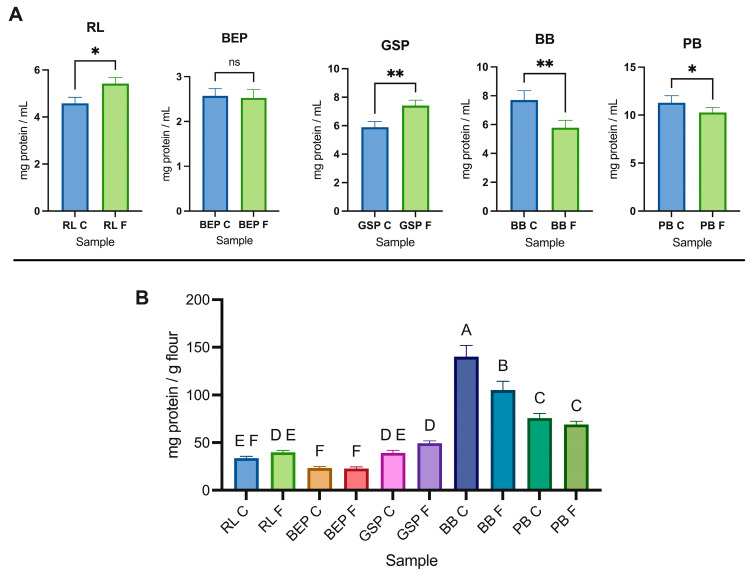
(**A**) Soluble protein concentration (mg/mL) in fermented and control (no Lp299v) pulse homogenates; ns (not statistically different) = *p* ≥ 0.05, * *p* ≤ 0.05, ** *p* ≤ 0.01, (**B**) Soluble protein concentration (mg/g of whole pulse flour). RL = red lentil, BEP = black eyed pea, GSP = green split pea, BB = black bean, PB = pinto bean, F = fermented, C = control. Different letters mean statistical difference between means (Tukey’s test, *p* ≤ 0.05).

α-Amylase hydrolyzes the α-1,4-glycosidic bonds in starch, and starch granules are surrounded by proteins known as starch-granule-associated proteins (SGAPs), which can alter the digestibility of starch [55,56]. Starch and protein interactions have been studied mainly in the context of starch retrogradation. Scott et al. [57] suggested that proteins can either promote or inhibit starch retrogradation depending on factors, such as the exposed protein residues and the protein-to-starch mass ratio. Additionally, the formation of hydrogen and covalent bonds between starch and proteins, as well as the high surface hydrophobicity of proteins, may repel water from starch granules upon protein aggregation. Proteins may also bind α-amylase, making it unavailable for starch hydrolysis [58]. As shown in Figure 6, the soluble protein of BB and PB decreased significantly (*p* ≤ 0.05) after fermentation. Interestingly, based on the optimization results, BB was the pulse with the lowest flour concentration, implying the presence of fewer saccharides and amino acids as carbon and nitrogen sources for bacterial growth and metabolism [48]. This reduction could support the assumption of smaller-peptide formation during fermentation. Protein degradation, particularly in albumins and glutenins, is influenced by the strain’s capacity to release proteases; in addition, a low pH level affects protein hydrolysis [59]. However, Gänzle [60] proposed that a pH drop shifts lactic acid metabolism, causing microorganisms to prefer utilizing amino acids over carbohydrates as their primary nutrient source. Furthermore, microbial activity during fermentation induces protein denaturation and changes in the pH, which contributes to structural alterations in proteins and amino acids and the generation of cross-links that may decrease their solubility [61]. The mild pasteurization process employed reduces endogenous bacteria, which could also impact digestibility or solubility.

In contrast to the decrease observed in BB and PB, RL and GSP exhibited a significant increase in protein solubility after fermentation, whereas BEP did not show a meaningful change in the protein concentration. Similarly, as presented in a study of quinoa and chickpea flour-blended beverages fermented with *L. acidophilus,* there was no change in the total protein concentration according to the Kjeldahl method. Nevertheless, when the authors used the Bradford method for soluble protein after fermentation, there was a significant increase in protein [40]. Therefore, if protein solubility is increased for RL and GSP during fermentation, proteins were hydrolyzed into short peptides, or starch was degraded, reducing their interaction with proteins and increasing protein solubility [62]. As presented by Liu et al. [63], the soluble protein concentration in a chickpea milk-like-beverage during fermentation with LBP HLJ29L tends to constantly increase over time. However, as demonstrated in the solid-state lactic acid fermentation of peas, protein solubility depends on the pH used during protein extraction [64].

### 3.6. DPP-IV and AG Inhibition Under Optimal Fermentation Conditions

The results of DPP-IV and AG inhibition are presented in Figure 3. There was no observed difference in AG inhibition in BB between the control (no Lp299v) and fermented groups. Similarly, no change was noted in DPP-IV inhibition in RL and GSP between the two conditions. However, AG inhibition was significantly increased in the case of RL and GSP, while DPP-IV inhibition showed a significant increase (*p* ≤ 0.05) only for BEP. Previous studies have shown variations in results regarding the inhibitory potential of diabetes-related enzymes when they go through a process of cooking, germination, hydrolysis, or fermentation [34,65,66]. It seems that the potential to inhibit different enzymes, such as DPP-IV, α-amylase, and AG, also depends on the protein- or peptide-extraction method and digestibility efficiency [67]. The results are compared to those obtained by Di Stefano et al. [68], who demonstrated a significant decrease in DPP-IV inhibition following heat treatment and solid-state fermentation in pulses, such as fava beans, green peas, and kidney beans. After fermentation, they also obtained significantly lower AG-inhibition activity in chickpeas, fava beans, kidney beans, and yellow peas. However, after simulated gastrointestinal digestion, green lentils fermented with LBP showed enhanced DPP-IV-inhibitory activity in intestinal Caco-2 cells [68]. In addition, other compounds soluble at pH 9.0 could affect or interfere with the assay, or the strain could not produce bioactive compounds with high DPP-IV and AG inhibition. Accordingly, the DPP-IV-inhibition capacity of LAB varies with the strain. As noted by Bhatia et al. [69], this variation may be due to the bacterial enzyme X-prolyl-dipeptidyl-amino-peptidase (PepX), a proline-specific peptidase that shares similarities with DPP-IV and exhibits similar enzymatic activity. In addition, dietary starch was partially or fully hydrolyzed in simple sugars by the α-amylase added before pasteurization; however, the resistant starch remaining in the solution theoretically affects glucose metabolism [70]. It was mentioned that anti-diabetic agents, including those targeting DPP-IV and AG inhibition, may not always effectively control blood glucose levels due to their limited efficacy and potential contraindications.

### 3.7. Correlation Analysis

**Figure 7:**
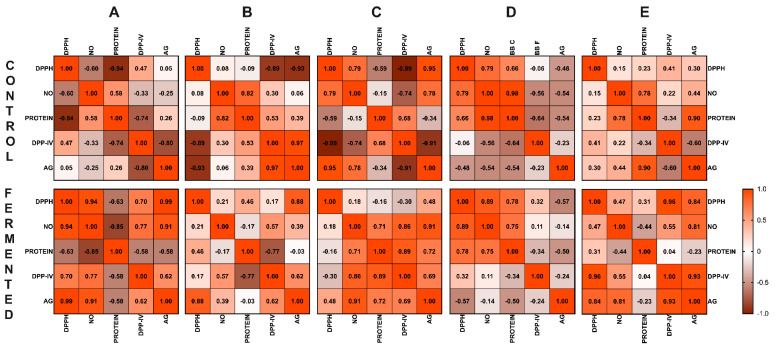
Heat map of Pearson correlation coefficient matrix for each pulse. (**A**) RL = red lentil, (**B**) BEP = black eyed pea, (**C**) GSP = green split pea, (**D**) BB = black bean, (**E**) PB = pinto bean. The color intensity represents the correlation strength: orange, strong positive correlations (r value close to 1.0, *p* ≤ 0.05); brown, strong negative correlations (r value close to −1.0, *p* ≤ 0.05); lighter shades, weaker correlations. DPPH = 2,2-diphenyl-1-picrylhydrazyl; NO = nitric oxide, DPP-IV = dipeptidyl peptidase IV inhibition, AG = α-glucosidase inhibition.

Figure 7 illustrates the Pearson correlation coefficient matrix for each pulse. The color intensity represents the relationship strength: orange indicates strong positive correlations (with an r value close to 1.0, *p* ≤ 0.05), brown denotes strong negative correlations (with an r value close to −1.0, *p* ≥ 0.05), and lighter shades represent weaker correlations; there were significant changes between controls and fermented pulses.

In the fermented RL product, as the scavenging capacity of DPPH increased, both the AG-inhibition and NO-scavenging capacity also increased, showing strong positive correlation between DPPH and AG (r = 0.99) and between DPPH and NO (r = 0.94). Additionally, NO exhibited a strong positive correlation with the inhibition of the enzymes DPP-IV (r = 0.77) and AG (r = 0.91). In the fermented BEP, a strong positive correlation was observed between DPPH and AG (r = 0.88), and DPP-IV showed a moderate positive association with NO (r = 0.57) and AG (r = 0.62). In the fermented GSP, there were strong positive interactions between NO and AG (r = 0.91), between NO and DPP-IV (r = 0.86), and between protein and DPP-IV (r = 0.89). In the fermented BB, a strong positive relationship was seen between DPPH and NO (r = 0.89), and NO also showed a moderate correlation with protein (r = 0.71). The fermented PB showed a strong positive interaction between DPP-IV and DPPH (r = 0.96), and AG showed strong positive correlations with DPP-IV (r = 0.93), NO (r = 0.81), and DPPH (r = 0.84).

### 3.8. In Vitro Caco-2 Cells Studies

**Figure 8:**
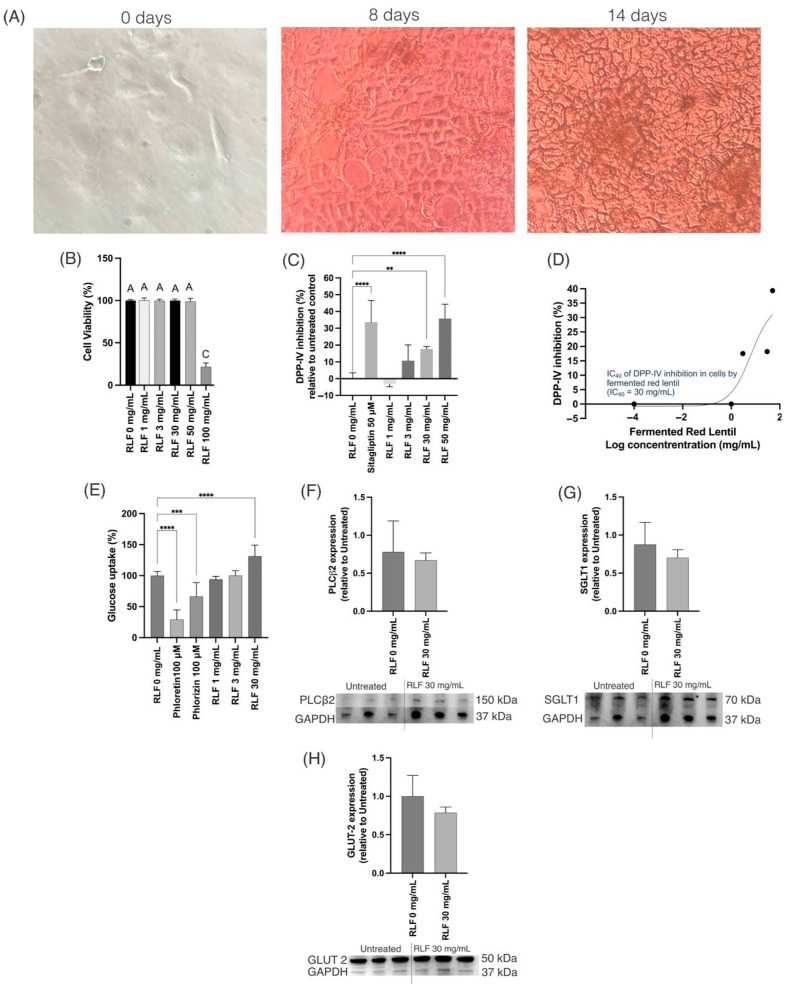
In vitro screening under optimized condition of fermented red lentil (RLF) in Caco-2 cells. (**A**) Cell differentiation process shows days 0, 8, and 14 out of 21. (**B**) Cell viability. Different letters in (**B**) mean statistical differences (*p* ≤ 0.05); cell viability was statistically affected by RL F at 100 mg/mL. (**C**) DPP-IV-inhibitory activity. (**D**) IC_40_ based on DPP-IV inhibition. (**E**) Glucose uptake. (**F**) PLCβ2, (**G**) SGLT1, and (**H**) GLUT 2 in differentiated Caco-2 cells. (**C**,**E**) ** *p* ≤ 0.01, *** *p* ≤ 0.001, and **** *p* ≤ 0.0001. (**F**–**H**) *p* > 0.05.

RL was tested in vitro on differentiated Caco-2 cells (Figure 8A) since it was the pulse with the highest soluble protein concentration after fermentation. The treatment with the freeze-dried fermented RL F at a concentration of up to 50 mg/mL was not cytotoxic (Figure 8B). The DPP-IV-inhibitory (Figure 8C) activity was different (*p* ≤ 0.01) from that of the control starting at a concentration of 30 mg/mL with an inhibitory IC_40_ concentration of 30 mg/mL (Figure 8D). DPP-IV is a membrane-bound peptidase that is expressed and located on the surface of different cells, such as Caco-2, and in the bloodstream [71]. DPP-IV inhibition is one of the options for T2D management since it cleaves glucagon-like peptide 1 (GLP-1) and glucose-dependent insulinotropic peptide (GIP), inducing the inactivation of these incretins [72,73]. The inactivation of these incretins will affect insulin secretion by binding to the GIP receptor and GLP-1 receptor in pancreatic β cells. Therefore, DPP-IV inhibition increases insulin secretion [74,75] and treatment with RL F likely results in improved insulin secretion, since it induced DPP-IV inhibition.

On the other hand, RL F at 30 mg/mL increased the absorption of glucose (Figure 8E). Since the transporters Glut2 and SGLT1 are the most significant glucose transporters in the gut [76], their expression was tested after RL F treatments. However, RL F showed a non-significant slight decrease in phospholipase C β2 (PLCβ2), sodium-glucose cotransporter (SGLT) 1, and glucose transporter (Glut) 2 (Figure 8F–H). The activation of the PLCβ2 pathway increases the expression of Glut 2, SGLT1, and SGLT2, increasing intestinal glucose transport [77]. Taken together, the screening results suggest that the observed bioactivity related to the T2D markers is primarily attributed to DPP-IV inhibition rather than glucose transporter expression. Thus, other markers in cells, such as HepG2 or differentiated 3T3-L1, should be tested considering the DPP-IV inhibition.

### 3.9. General Observations

Various changes in physical and sensory properties were observed after the incubation of the fermented and control (no Lp299v) pulses. For example, the texture of the fermented pulses appeared less viscous, possibly due to the hydrolysis of resistant starch by Lp299v, which may have targeted different bonds other than the α-1,4-glucosidic bonds previously hydrolyzed by α-amylase [78]. In addition, there was some proteolytic activity before fermentation due to the purity of the commercial α-amylase used, which was found to contain trace amounts of proteolytic enzymes. Color differences were also noted between fermented and control pulses. The fermented RL appeared slightly lighter in tone than its control, while the fermented BB exhibited a red–purple hue compared with the black–purple color to the control BB. These changes could be attributed to pH variations affecting pigments, such as flavonoids [79]. Aroma differences were also perceived, with the fermented pulses developing a slightly acidic or sour scent. Additionally, they exhibited increased foaming when mixed or agitated, likely due to the presence of the low-molecular-weight proteins, carbohydrates, and saponins liberated by bacterial activity [80]. The lower pH may have further contributed to the enhanced foaming capacity of the fermented pulses [81]. The observations made in this studysuggest that fermentation induces notable changes in the physicochemical properties of pulses, which may influence their functional behavior. Further research is warranted to elucidate the mechanisms underlying these transformations, including the specific roles of microbial activity and compound conversion during fermentation.

## 4. Conclusions

The pulse homogenate provided a suitable environment for the selected strain, as indicated by the acidification observed after fermentation. Optimized lactic acid fermentation with Lp299v selectively enhanced the antioxidant and antidiabetic potential of the pulse homogenate, depending on the pulse substrate. Notably, GSP and RL exhibited the greatest improvements in bioactivity, including enhanced DPPH scavenging, increased protein solubility, and greater α-glucosidase inhibition. These findings highlight the potential of fermentation as a strategy for improving the functional properties of pulses, supporting their development as functional food ingredients with health-promoting attributes.

## Figures and Tables

**Table 1:** The matrix of the experimental data, final pH, and DPPH-scavenging capacity of fermented pulses according to the Box–Behnken design.

Factor			Final pH					Response: DPPH-Scavenging Capacity (%)				
X_1_: Time	X_2_: Bacteria	X_3_: Flour Concentration	RL	BEP	GSP	BB	PB	RL	BEP	GSP	BB	PB
−1	−1	0	3.92	4.10	3.97	4.28	4.03	52.33	66.62	55.06	68.28	63.92
1	−1	0	3.76	3.80	3.75	3.88	3.75	45.42	59.44	40.96	59.99	60.11
−1	1	0	3.85	3.83	3.80	4.06	3.81	55.44	65.11	48.29	69.06	64.20
1	1	0	3.71	3.70	3.70	3.88	3.79	50.26	60.30	37.69	59.92	57.76
−1	0	−1	3.85	3.87	3.82	4.08	3.85	30.92	39.34	23.67	62.38	36.84
1	0	−1	3.80	3.88	3.75	3.98	3.80	30.92	35.61	22.79	53.87	29.92
−1	0	1	3.88	3.92	3.91	4.05	3.97	60.10	69.63	72.99	57.45	79.57
1	0	1	3.76	3.79	3.77	3.94	3.85	52.16	47.45	38.41	57.81	72.51
0	−1	−1	3.84	3.86	3.68	3.99	3.77	28.67	36.25	25.50	57.95	36.77
0	1	−1	3.83	3.79	3.74	3.99	3.74	32.64	36.90	22.95	57.24	30.82
0	−1	1	3.66	3.83	3.81	4.03	3.88	56.13	55.64	52.43	52.88	70.43
0	1	1	3.83	3.79	3.78	3.96	3.87	50.26	46.59	55.94	46.20	76.80
0	0	0	3.92	3.77	3.74	3.88	3.85	49.91	51.62	37.61	58.37	62.26
0	0	0	3.92	3.78	3.76	3.92	3.81	53.71	53.91	38.01	57.67	57.27
0	0	0	3.92	3.77	3.75	3.92	3.82	55.61	58.29	41.27	54.92	59.28
0	0	0	3.92	3.77	3.76	3.90	3.83	54.06	59.80	45.74	57.38	57.76
				Fit statistics								
				Standard deviation				2.43	4.01	4.45	3.00	1.90
				Mean				47.41	52.66	41.21	58.21	57.26
				Coefficient of variance (%)				5.12	7.61	10.80	5.17	3.32
				R^2^				0.98	0.95	0.94	0.88	0.99
				Adjusted R^2^				0.95	0.87	0.90	0.70	0.98
				Predicted R^2^				0.81	0.51	0.74	−0.73	0.96
				Adeq precision				17.27	11.26	16.92	8.00	32.32

Note: Time: −1 = 8 h, 0 = 16 h, 1 = 24 h; Bacteria: −1 = 0.25 mL (~5 × 10^8^ CFU/mL), 0 = 1 mL (~2 × 10^9^ CFU/mL), 1 = 1.75 mL (~3.5 × 10^9^ CFU/mL); Flour concentration: −1 = 3%, 0 = 9%, 1 = 15%. RL = red lentil; BEP = black eyed pea; GSP = green split pea; BB = black bean; PB = pinto bean. All pulses had a pH adjusted to 6.9 before hydrolysis. The final pH of the controls (no Lp299v) was RL = 5.89 ± 0.26, BEP 5.51 ± 0.20, GSP = 5.40 ± 0.11, BB = 5.66 ± 0.15, and PB = 5.76 ± 0.34.

**Table 2:** ANOVA of RSM *p*-value for fermented pulses.

	*p*-Value				
	RL	BEP	GSP	BB	PB
**Model**	0.000	0.003	0.000	0.034	0.000
**X_1_-Time**	0.027	0.016	0.001	0.024	0.004
**X_2_-Bacteria**	0.413	0.455	0.489	0.462	0.769
**X_3_-Flour concentration**	0.000	0.001	0.000	0.091	0.000
**X_1_ X_2_**	0.734	0.777	0.703	0.892	0.515
**X_1_ X_3_**	0.153	0.061	0.004	0.191	0.972
**X_2_ X _3_**	0.089	0.272	0.513	0.359	0.018
**X_1_^2^**	0.735	0.032	-	0.009	0.128
**X_2_^2^**	0.146	0.511	-	0.370	0.504
**X_3_^2^**	0.000	0.000	-	0.016	0.001
**Lack of Fit**	0.495	0.435	0.376	0.072	0.746

Note: ANOVA quadratic and two-factor interaction fit model for RL = red lentil, BEP = black eyed pea, BB = black bean, PB = pinto bean. Two factor interaction fit model for GSP = green split pea (- indicates no value for X_1_^2^, X_2_^2^, and X_3_^2^). Statistical difference = *p* ≤ 0.05 and not statistically different = *p* ≥ 0.05.

## Data Availability

The original contributions presented in this study are included in the article. Further inquiries can be directed to the corresponding author.

## Supplementary Materials

The following supporting information can be downloaded at: https://www.mdpi.com/article/10.3390/antiox14050523/s1, Figure S1: Representative results from LC-ESI-MSMS showing the peaks of fermented pulses in comparison to the control (no Lp299v).

## Abbreviations

RL, red lentil; BEP, black eyed pea; GSP, green split pea; BB, black bean; PB, pinto bean; DPPH, 2,2-diphenyl-1-picrylhydrazyl; CFU, colony forming unit; LAB, lactic acid bacteria; Lp299v, *Lactiplantibacillus plantarum* 299v; NO, nitric oxide; NO_2_, nitrite; AG, α-glucosidase; DPP-IV, dipeptidyl Peptidase-IV; T2D, type 2 diabetes.
