# Supplementary figures and images for: Optimized Fermentation Conditions of Pulses Increase Scavenging Capacity and Markers of Anti-Diabetic Properties
Source: Antioxidants (Basel). 2025 Apr 27;14(5):523. doi: 10.3390/antiox14050523 (PMC12108444; doi:10.3390/antiox14050523)

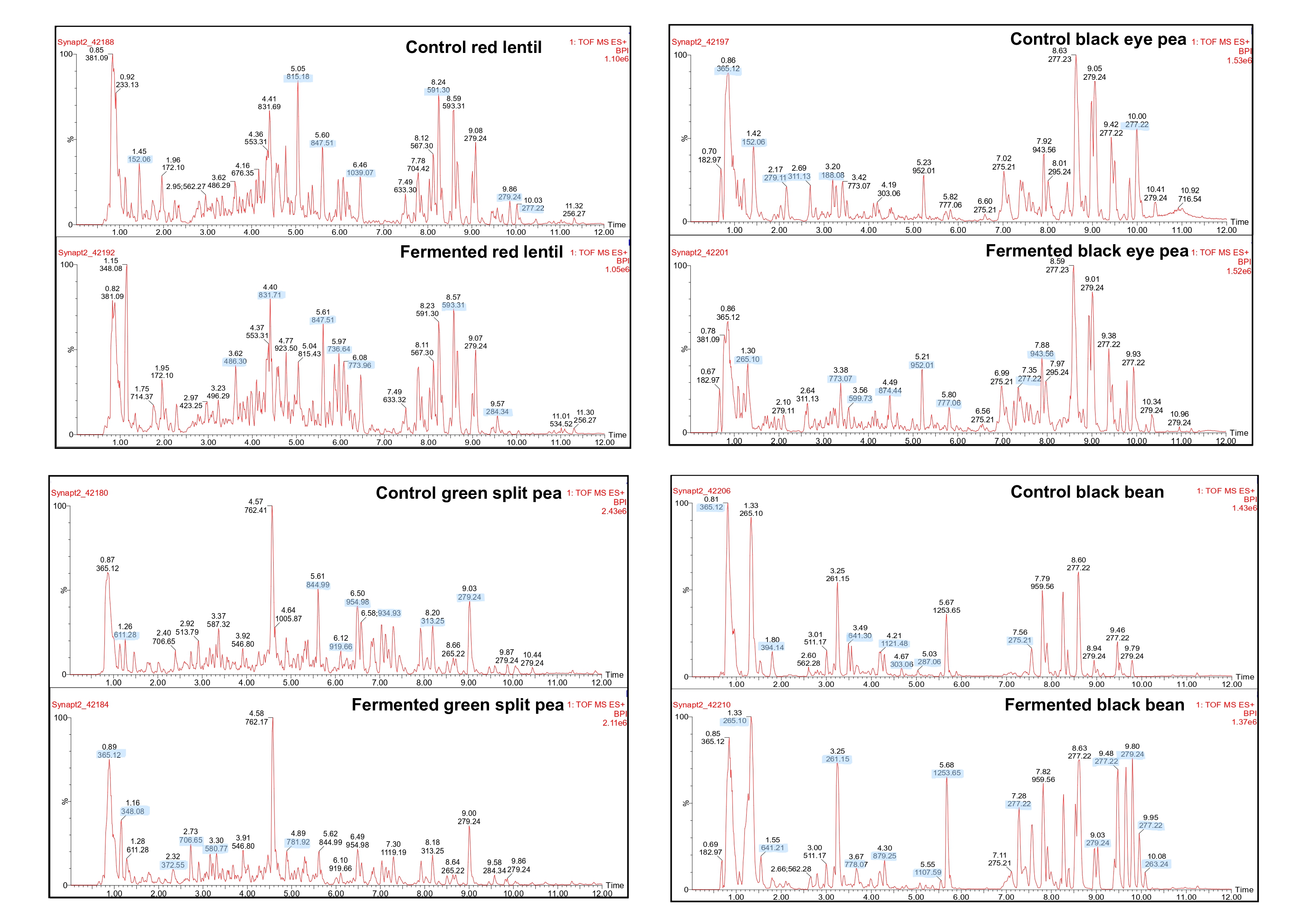

Supplement: Supplementary file 1 [file antioxidants-14-00523-s001.zip › antioxidants-3560889-supplementary.jpg]
